# Viscoelastic and Properties of Amphiphilic Chitin in Plasticised Polylactic Acid/Starch Biocomposite

**DOI:** 10.3390/polym14112268

**Published:** 2022-06-02

**Authors:** N. G. Olaiya, C. Maraveas, Mohamed A. Salem, S. Raja, Ahmad Rashedi, Abdullah Y. Alzahrani, Zeinhom M. El-Bahy, Funmilayo G. Olaiya

**Affiliations:** 1 Department of Industrial and Production Engineering, Federal University of Technology Akure, PMB 704, Akure 340110, Nigeria; 2 Farm Structures Laboratory, Department of Natural Resources Management and Agriculture Engineering, Agricultural University of Athens, 11855 Athens, Greece; 3 Department of Chemistry, Faculty of Science and Arts, King Khalid University, Mohail, Abha 63763, Saudi Arabia; masalem@kku.edu.sa (M.A.S.); ayalzahrani@kku.edu.sa (A.Y.A.); 4 Department of Chemistry, Faculty of Science, Al-Azhar University, Nasr City, Cairo 11884, Egypt; zeinelbahy@azhar.edu.eg; 5 School of Mechanical Engineering, Vellore Institute of Technology, Vellore 632004, India; raja.s2020@vitsstudent.ac.in; 6 College of Engineering, I.T. & Environment, Charles Darwin University, Ellengowan Drive, Casuarina 0810, Australia; mabrur.rashedi@cdu.edu.au; 7 School of Industrial Technology, Universiti Sains Malaysia, Penang 11800, Malaysia; phunmieoseyemi@gmail.com

**Keywords:** filler, plasticiser, viscoelastic, amphiphilic, biocomposite

## Abstract

The enhancement of the PLA thermomechanical properties is significant due to its suitability as a replacement for primary synthetic polymer use in diverse industrial production. The amphiphilic chitin was used as a compatibilizer in PLA/starch biocomposite. The properties of plasticised polylactic acid blended with starch, and amphiphilic chitin was studied for enhanced thermomechanical and viscoelastic properties. Chitin was modified using acetylated substitution reaction and blended with plasticised PLA/starch biocomposite. The biocomposite was prepared with combined compression and melt extrusion techniques. The biocomposite’s thermomechanical, thermal, mechanical, and morphological properties were studied using dynamic mechanical analysis, TGA-DSC, tensile test, and scanning electron microscopy. The storage and loss modulus were significantly enhanced with increased amphiphilic chitin content. Similarly, the single peak of tan delta showed good miscibility of the polymeric blend. Additionally, the modulus increases with frequency change from 1 Hz to 10 Hz. The thermal stability of the biocomposite was observed to be lower than the neat PLA. The tensile properties of the biocomposite increased significantly more than the neat PLA, with P4S4C having the highest tensile strength and modulus of 87 MPa and 7600 MPa. The SEM images show good miscibility with no significant void in the fractured surface. The viscoelastic properties of PLA were enhanced considerably with plasticizer and amphiphilic chitin with improved biodegradability. The properties of the biocomposite can be adapted for various industrial applications.

## 1. Introduction

Biopolymers based on lactic acid (PLA) are the most promising renewable polymers. Biopolymers are compostable, biocompatible, and easily processed with similar techniques used in conventional plastic manufacturing [1,2]. PLA thermomechanical properties enhancement is of utmost priority because of its similar processability with resins used in many industrial applications [3]. PLA can be synthesised directly from lactic acid or via ring-opening polymerization of lactide dimer, which is a cyclic diester of lactic acid derived from sugar feedstock fermentation. PLA is termed a chiral molecule because it exists in two stable forms: D-lactide and L-lactide. PLA is semicrystalline with both crystalline and amorphous parts [4]. However, PLA crystallinity is usually less than 10%. The crystallinity properties of PLA depend on the preparation method [5]. As an example, polymerization can alter the stereochemical structure of the monomer, resulting in amorphous or semicrystalline polymers [6,7].

PLA has been reinforced with other biopolymers, of which starch, chitin, and cellulose are the most abundant. Starch is a naturally occurring plant and animal product. Biopolymer is abundant and inexpensive [8,9]. Chitin is a non-toxic, biodegradable polymer with exceptional biocompatibility properties with the human system. It is prevalent in crabs, shrimps, crustaceans [10,11], and abundant biopolymers like cellulose [12]. Cellulose is majorly isolated from a plant source. However, starch and chitin are derived in abundance from non-wood sources that can be used without much more impact on the ecosystem than cellulose. 

Thus, starch has been used as reinforcement in PLA without impairing its biodegradability. Nevertheless, as the starch concentration of the biocomposite increases, the biocomposite’s tensile and elastic modulus properties have been found to decrease [13,14,15,16]. This has been linked to the two polymers’ molecular differences: starch is hydrophilic, whereas PLA is hydrophobic [17]. To obtain good miscibility while combining PLA, the literature stated that the ratio of polymer blend to reinforcing phase should be 9:1 by weight [18]. Previous research on PLA/Starch demonstrated that starch improves PLA biodegradability, but the miscibility is affected due to the hydrophilic nature of starch as opposed to hydrophobic PLA [16,19]. Gazzotti, Rampazzo [17] demonstrated that employing multifunctional alcohol (glycerol) as a plasticiser improves the PLA/Starch miscibility, but degrades the biocomposite’s tensile strength.

Additionally, Aranda-Garcia [20] noted that starch’s hygroscopic nature and impact strength decrease when the PLA weight percent increases. Nevertheless, the modulus was improved to a certain percentage of starch. The extrusion of PLA/Starch by Zuo et al. [18] reveals that the interfacial miscibility of Starch and PLA increased with a decrease in starch percentage. Their report concluded that the starch/PLA ratio of 1:10 had better properties. in another work, it was shown that starch had a reinforcing effect Móczó, Kun [15]. They worked with PLA/Starch and the Compression moulding process, utilizing glycerol as a plasticiser. Increased glycerol content increased and influenced stiffness and strength. However, agglomeration in PLA/starch biocomposite is still a challenge.

On the other hand, chitin has been employed to reinforce PLA with excellent dispersion but slightly improve tensile strength [21]. As a result, it was proposed that a low percentage of chitin nanofibres be combined with PLA to improve mechanical qualities. Chitin is widely used to improve the properties of PLA because of its abundance, low cost, and bioabsorbability. It has a variety of applications in packaging and aerogel development materials with excellent performance. 

PLA/Starch biocomposite has been reported with agglomeration and low tensile strength due to differences in the nature of the polymer. Several researchers have enhanced the miscibility between PLA and Starch due to their suitability for multiple applications. PLA is hydrophobic, and starch is hydrophilic. Therefore, in this study, amphiphilic chitin has both hydrophobic and hydrophilic ends that act as a nonreactive compatibilizer to enhance their miscibility and strength. This research explored a novel green approach to using amphiphilic chitin as a compatibilizer between PLA and Starch against complex chemicals. Amphiphilic chitin has been reported to be a non-toxic and biodegradable derivative of chitin. Chitin was modified to amphiphilic chitin using two-step acylation to incorporate both hydrophilic and hydrophobic functional groups. The use of two-step acylation in this study has not been reported before. Additionally, amphiphilic chitin as a compatibiliser in plasticised PLA/starch has not been researched. The amphiphilic chitin was filled in plasticised PLA/starch to form a biocomposite to improve its miscibility and enhance its strength. The use of amphiphilic chitin in a PLA-starch biocomposite has not been reported. Furthermore, this study develops a novel green approach to using amphiphilic chitin as a compatibilizer between PLA and starch to enhance its properties for industrial application.

## 2. Materials and Methods

### Materials

Polylactic acid, starch, and amphiphilic chitin are the biocomposite components. Nature Works (Minnetonka, MN, USA) supplied pelletized poly(lactic acid) (4032D) for this study. PLA (4032D) was used because of its outstanding resistance to oil, grease, twist, machinability, and dead fold properties. PLA melts between 150 and 170 degrees Celsius and has a glass transition temperature of 65 °C. The PLA has a tensile strength of 53.5 MPa, a modulus of 3500 MPa, and an impact strength of 2.99 kJ/m^2^. PLA (4032D) has a molecular mass of 100 kg/mole on average, a specific gravity of 1.24, and an MFR (melt flow rate) of g/10 min (2.16 kg, 210 °C). Chitin was commercially obtained in Sigma Aldrich, South Africa.

**Figure 1:**
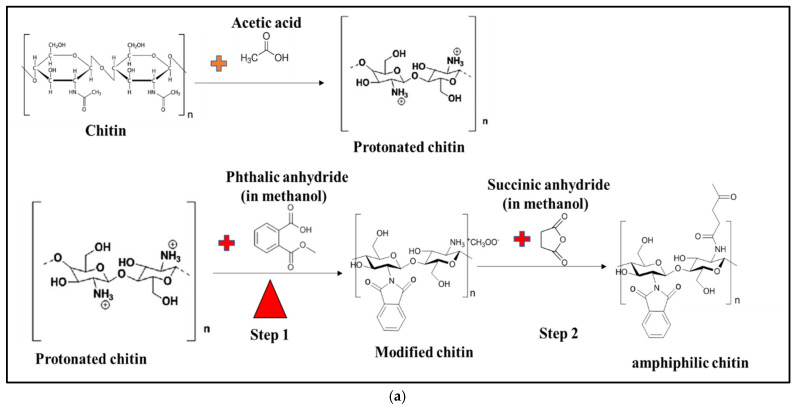

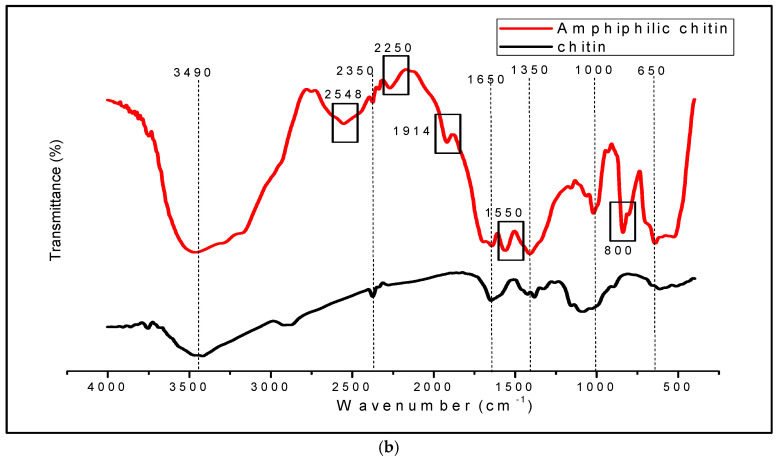
(**a**) Schematic chemical reaction of preparation of amphiphilic chitin. (**b**) FT-IR graph of amphiphilic chitosan and conventional chitosan.

Amphiphilic chitin was produced using a two-step acetylation reaction, as shown in Figure 1a. Chitin from crab (obtained from Sigma Aldrich, Malaysia) was dissolved in DMF acidified with HCl. Phthalic anhydride was added to the solution heated at 60 °C and stirred using a magnetic stirrer for 2 h. The mixture was neutralised with NaOH to remove excess acid, and phenol anhydride was added to complete the substitution reaction. The amphiphilic chitin was precipitated into a gel using NaHCO_3_ and filtered off. The gel was freeze-dried after washing in distilled water and grounded to powder.

PLA in pellets form was reduced to a smaller size of 0.045–0.71 mm with a granulator AA-150 power (Pulian, Taichung, Taiwan) to enhance miscibility. The ground particles were dried at 60 °C in a dryer Luxor 50 (Motan, Überlingen, Germany) for 4 h and then dissolved in chloroform (plasticiser). The amphiphilic chitin and starch of varying compositions were mixed with PLA solution in a Thermo Fisher Scientific Rheomixer 03 thermoelectric (Waltham, MA, USA). The polymer blend was extruded to filament with a Thermo Fisher Scientific twin-screw extruder Process 11 (Waltham, MA, USA) at a temperature profile of 120–170 °C and quenched in water. The filament was pelletized with a Thermo Fisher Scientific pelletizer 11 (Waltham, MA, USA), and Carver pressed into characterisation shapes for 15 min using a compression moulding machine (Carver, Wabash, IN, USA) at 10 MPa, 170 °C. The percentage of PLA in the polymer blend composite is highest in the matrix. The test samples (with composition as shown in Table 1) were stored in Ziploc bags.

Dynamic mechanical analysis (DMA), tensile testing, thermogravimetry analysis (TGA and DTG), differential scanning calorimetry (DSC), and scanning electron microscopy were used to characterise the thermomechanical, mechanical, thermal, and morphological properties, respectively. The thermomechanical characteristics of materials were determined using dynamic mechanical analysis over a temperature and frequency range. This was accomplished with the assistance of a DMA 8000 PerkinElmer Inc. (Columbus, OH, USA). The shape and dimensions of the samples were determined using the ASTM D4065 for polymer composites at −50 to 150 °C for 1 to 50 Hz. The change in modulus storage (E′), loss (E″), and loss factor (tan δ) were obtained.

The thermal characterisation of the biocomposite was determined using a TG-IR-GCMS thermogravimetric analyser (PerkinElmer Inc., Columbus, OH, USA). The thermogravimetric test was conducted in accordance with ASTM E1131, 15–20 mg of sample, at 10 °C/min from ambient temperature to 600 °C in air. Origin software was used to analyse and plot the data (Pro 8.1). Additionally, differential scanning calorimetry was used to analyse the material’s behaviour as a function of temperature change using a DSC model 6 (Perkin–Elmer, Schwerzenbach, Switzerland, ASTM 3418). Temperatures of the glass transition, crystallisation, and melting were measured. The samples used had a mass of between 5 and 7 mg. Tensile testing was performed on a 20 kN load using an Intron Universal Testing Machine (model 5966) (Instron, Norwood, MA, USA). The crosshead speed was kept constant at 2 mm/s. Biocomposite samples with a cross-sectional size of 5 mm by 10 mm and a gauge length of 30 mm were created and submitted to the American standard (ASTM) for materials testing. The tensile test is performed in accordance with ASTM D3039. The microstructure of the tensile fractured surface of the biocomposite was examined with a scanning electron microscope (SEM). SEM was utilised to analyse the microstructure after the gold-coated material using secondary electron (S.E.) and backscattered electron (BSE) signals. The SEM was operated at a 2.00 kV SEI accelerating voltage.

A soil burial test was also used to determine the material’s biodegradability qualities. The samples were cut into 2 cm × 2 cm squares and buried 10 cm into organic soil for 150 days. The pre-weighed PLA and biocomposite samples were weighed before burial and again after one month to determine the deterioration rate. The samples are removed and rinsed with distilled water before being dried in an oven for 24 h at 40 degrees Celsius before being weighed. Previous research and ISO 846 (Plastics-Evaluation of Microorganisms’ Action) were used to conduct the soil burial test. The relative humidity of the compost soil was kept between 40 and 50 percent at room temperature, and the soil was injected with distilled water on a regular basis to keep it moist enough for microbial activity. The weight loss was calculated from the percentage difference between the final and the initial weight of the material at 0, 100 and 150 days.

Water absorption was conducted to measure the wettability of the composite based on ASTM D570. The samples were pre-weighed and immersed in water for 24 h, and the initial and final weight differences were recorded. A dimension was 1 cm by 1 cm by 3 cm was used for all samples.

## 3. Results

### 3.1. Properties of Chitin and Amphiphilic Chitin

Figure 1b shows the FT-IR analysis of chitosan and modified chitin. Unmodified and modified chitin showed a band between 3100 and 3500 cm^−1^, indicating O.H. and N.H. stretches, respectively. On the unaltered chitin, a minor peak was found between 2800 and 2930 cm^−1^, indicating aldehyde -C-H stretches. Additionally, from the amino group’s amide functional group and acetylation, a brief peak at 1600–1680 cm^−1^ is referred to as C=O and -C=C-. The alkene sp^2^ C-H bends are 1550 cm^−1^ (CO_2_^−^), 1400 cm^−1^ band sp3 -C-H bend, 1154 cm^−1^ C-O-C bridge, 800 cm^−1^, and 650 cm^−1^. The existence of 800 cm^−1^ bands in amphiphilic chitin indicated the presence of an alkene C-H bend, while an increase in 650 cm^−1^ bands indicated the presence of a C-H bond, indicating the presence of an alkyl group. Changes in the bands 1500 cm^−1^ and 800 cm^−1^ and the formation of new peaks are typical of alkyl group attachment (substitution) to the chitin chain. The alkyl group attachment occurred in the amine functional group present in the chitin in previous research on this modification method [22]. During the alteration, the hydrogen ions of -NH_2_ were replaced [23]. Figure 1a shows the chemical reaction equation for the modification procedure. The schematic drawing was confirmed based on previous literature on the preparation of amphiphilic chitin [24]. According to their findings, the amine functional group, not the hydroxide, was the active location for substitution reactions [25,26].

### 3.2. Properties of Neat PLA and Biocomposite

**Figure 2:**
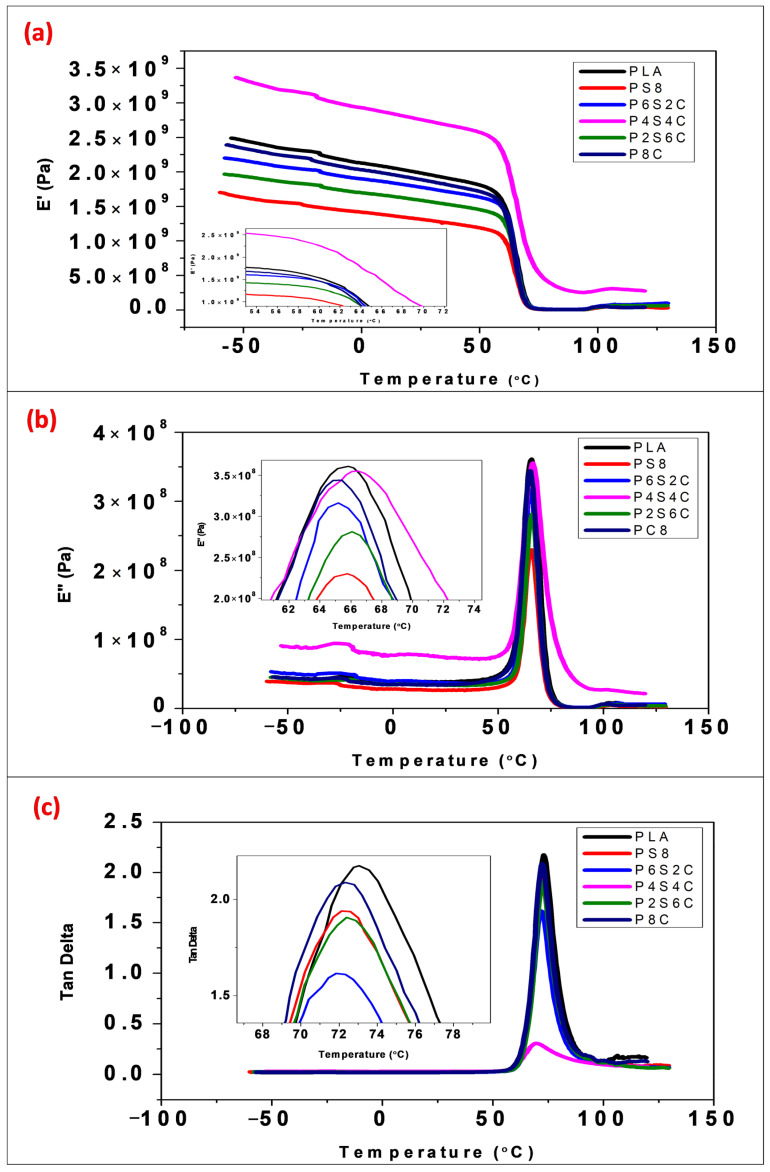
DMA curves at 0.1 Hz of the neat PLA and biocomposite composites (**a**) storage modulus (E′); (**b**) loss modulus (E″), and (**c**) loss factor (tan δ).

This DMA is an adaptable thermomechanical analysis technique that is frequently used to analyse polymeric composites’ viscoelastic and structural behaviour to identify their relevant damping and stiffness properties for various applications. Periodic stress or strain deforms the materials [27]. DMA was used in this study to observe sensitive matrix transitions, relaxation processes, and the form of composites [28]. When investigated across a wide range of frequencies and temperatures, the DMA of polymeric composites is extremely valuable. The dynamic storage modulus (E′) is the most crucial metric for determining a biocomposite material’s load-bearing capacity. The influence of temperature on the storage modulus of pure PLA and biocomposite blends is illustrated in Figure 2a.

The storage modulus of the biocomposite (E′) lowers progressively with an increase in temperature and more significantly as the biocomposite enters the glass transition area owing to the entire polymer chain’s free mobility [29]. The E′ value increased with increased amphiphilic chitin and reduced starch content. This can be explained based on the nature of the blend in the matrix. PLA being hydrophobic, has good miscibility with amphiphilic chitin, which results in increased modulus value with an increase in amphiphilic chitin content, while starch is hydrophilic, reducing its miscibility with PLA matrix [26,30]. Additionally, as seen in Figure 2, there is a substantial variation in the glassy region between the P4C4S biocomposite and other biocomposites. However, P4C4S was observed to have the highest storage modulus, which probably indicates a neutralising compatibility effect arising from the presence of both fillers in equal amounts. This may be due to the compatibilizer effect of amphiphilic chitin, which enhanced the miscibility of starch of equal percentage. The previous report shows that chitin shows slight miscibility with starch due to the presence of the hydroxide functional group despite its being hydrophobic [31,32].

The rate of reduction of E′ value for both biocomposites and neat PLA across the glass transition area is identical. This is probably due to the hydrodynamic effects of the filler material introduced into the PLA matrix and mechanical constraints on the PLA matrix’s mobility and deformability [33]. Similar observations have been made elsewhere [34,35]. This also implies that the amphiphilic chitin-starch blend has a more significant effect on E′ below than above the glass transition temperature. To investigate the relationship between the insertion of filler and the matrix. In Equation (1), a coefficient C denotes the filler’s efficiency on the E′ of biocomposites [36,37].

(1) $$C=\frac{C_{comp}}{C_{resin}}$$

where $C_{comp}=\left(\frac{{E^{\prime }}_{g}}{{E^{\prime }}_{r}}\right)$ is the ratio of the storage modulus at 25 °C (*E′_g_* before glassy region) and 130 °C (*E′_r_* before rubbery region) of the composite. Additionally, $C_{resin}=\left(\frac{{E^{\prime }}_{g}}{{E^{\prime }}_{r}}\right)$ is the ratio of the storage modulus at 25 °C (*E′_g_* before glassy region) and 130 °C (*E′_r_* before rubbery region) of the matrix. 

The value of *C* is inversely proportional to the filler’s effectiveness [37]. Table 2 presents the coefficient of filler efficiency for the biocomposite. 

The lowest was obtained for biocomposite with P4S4C, and the highest was P8C. Therefore, this shows that the combined starch–amphiphilic chitin addition has more effectiveness than amphiphilic chitin or starch used separately. These coefficient values justify the increased storage modulus results and the combined use of starch and amphiphilic chitin fillers. This can be scientifically explained as a bridging effect of amphiphilic chitin having a compatibiliser effect due to its double functional group (i.e., Hydroxide and amine), allowing its bonding with starch and PLA.

The loss modulus gives the energy lost per deformation cycle of a system at constant strain or stress amplitude [38]. The loss modulus with a temperature change of neat PLA and biocomposites is shown in Figure 2b. The E″ of neat PLA and biocomposites reaches a maximum at the glass transition temperature and follows the same pattern as the storage modulus E′ above this point. Below the glass transition temperature, the blend’s effect on E″ is discernible and negligible above it. When the amphiphilic chitin concentration is increased below the glass transition temperature, the E″ increases and vice versa with starch, most likely due to changes in polymeric mixes [30]. However, a differentiating value was detected for samples with equal amphiphilic chitin and starch percentage (P4S4C).

Tan δ is a damping term that refers to the impact resistance of a material [31]. As the damping peak occurs at the glass transition temperature, the point at which the material changes from rigid to elastic, it has to do with the movement of tiny groups, segments, and chains within the polymer structure, which are initially frozen in place and begin to move at the glass transition temperature [32]. The use of fillers alters the damping properties of biocomposite materials due to a change in the shear stress distribution, which depends on the filler concentration and type utilised. The dissipation of viscoelastic energy in the matrix material is related to mixing in a biocomposite. The elastic properties of the filler material have a significant effect on the damping qualities of the biocomposite. Figure 2c illustrates the effect of temperature on the tan δ of neat PLA and biocomposites. The tan curves exhibit a maximum at the glass transition, comparable to the E′ and E″ curves. Tan δ peak biocomposites show a single peak at a lower temperature than neat PLA, which signifies good interaction between the polymer blends. The interaction between the filler and the PLA matrix may decrease as the amphiphilic chitin and starch level increases above 8%. PLA and chitin have been reported with an agglomerate above 10% [22,33]. The fact that the peak value of the tan decreases when the mix contains more amphiphilic chitin and starch indicates the biocomposite’s stability. Biocomposites exhibit superior interface bonding than pure PLA since a biocomposite with poor interface bonding loses more energy than one with adequate interfacial bonding [34]. Sample P4S4C was observed to have the lowest tan δ peak, which corroborates its high storage modulus.

Temperature, time, and frequency influence a material’s viscoelastic properties [37]. This is because the material’s molecular structure has been reorganised in order to alleviate localised stress. Modulus measurements taken at higher frequency would probably yield higher values than low frequency [39,40]. The storage modulus, loss modulus, and loss factor were also investigated in this study over a range of frequencies to observe this effect.

**Figure 3:**
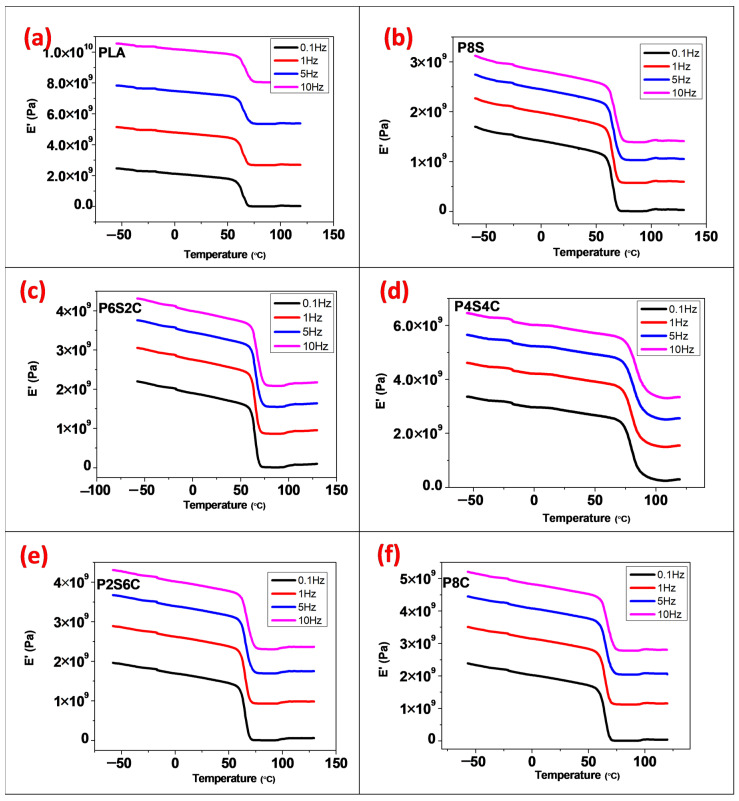
The effect of frequency on DMA storage modulus curves of the (**a**) PLA, (**b**) P8S, (**c**) P6S2C, (**d**) P4S4C, (**e**) P2S6C and (**f**) P8C biocomposite.

**Figure 4:**
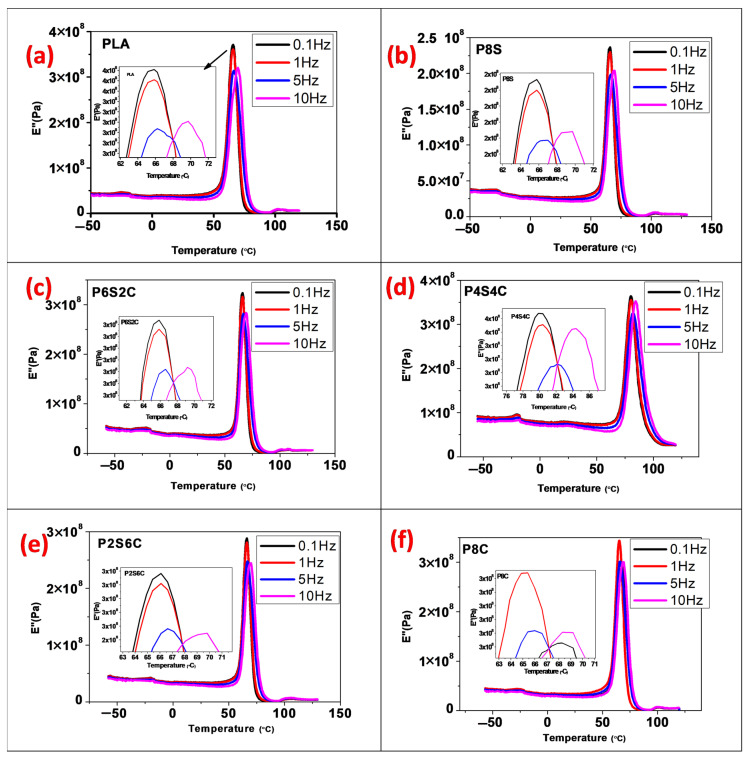
The effect of frequency on DMA loss modulus curves of the (**a**) PLA, (**b**) P8S, (**c**) P6S2C, (**d**) P4S4C, (**e**) P2S6C and (**f**) P8C biocomposite.

**Figure 5:**
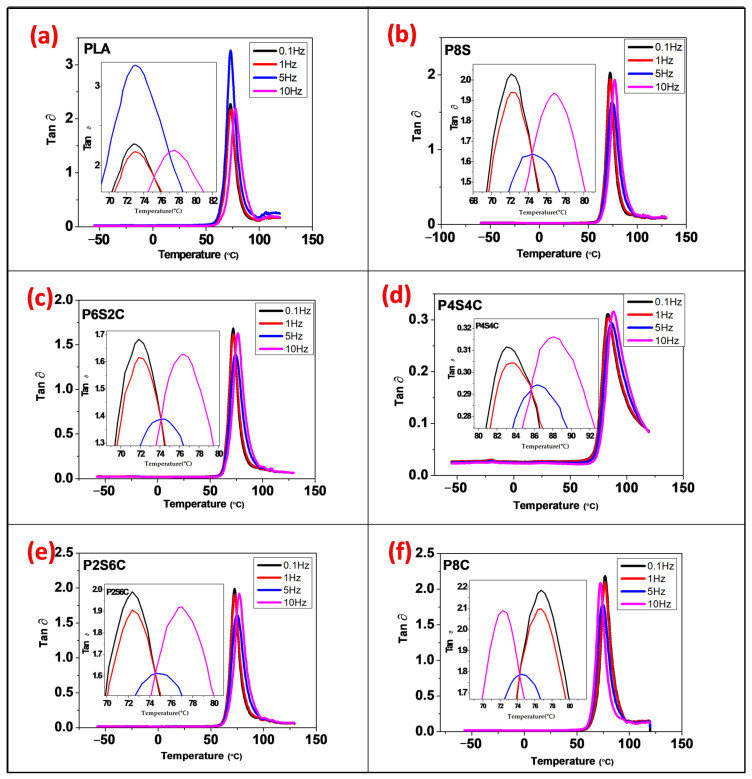
The effect of frequency on DMA loss factor curves of the (**a**) PLA, (**b**) P8S, (**c**) P6S2C, (**d**) P4S4C, (**e**) P2S6C and (**f**) P8C biocomposite.

The graph of storage modulus, loss modulus, and dissipation factor are displayed in Figure 3, Figure 4 and Figure 5 for frequencies 1–10 Hz. DMA observations were taken over a short period, and higher values were observed (high frequency, 10 Hz) for all samples. Figure 3a–f demonstrate the fluctuation of E′ with the frequency of the neat PLA and biocomposite samples. The storage modulus value was observed to uniformly increase at both the glassy and rubbery region from 1 Hz to 10 Hz. It was discovered that increasing the gradual increase in frequency raises the E′ values significantly and becomes more pronounced at 10 Hz. 

The effect of frequency on the loss modulus (E″) of the tidy PLA and biocomposite is shown in Figure 4a–f. With increasing frequency, the apex of the loss modulus curve tends to shift to a higher temperature, which means that the material performs better at a higher frequency than lower frequency. Similar to the storage modulus, a sudden trough in the loss modulus and rise shows the material damping response probability. Figure 5a–f show the loss factor values for the neat PLA and biocomposite over various frequencies. With increasing frequency, the tan peak likewise shifts to a greater temperature. As observed in the storage and loss modulus, the tan graph also showed a sudden trough.

Table 3 summarises the T_g_ values of all the samples with varying frequencies. Frequency, temperature, and time all affect a material’s viscoelastic characteristics. If material is subjected to constant stress, its elastic modulus will decrease. This is due to the material’s molecular rearrangement, which reduces localised stress. Modulus measurements made over a short period (high frequency) yield higher results, whereas measurements taken over a long period (low frequency) yield lower results [41,42]. The T_g_ of all samples significantly increases with increasing frequency. The increase in the Tg values means that the relaxation movement of PLA chains is delayed at high frequency. High frequencies can induce more elastic-like behaviour. If the frequency is chosen to be high enough, a material will behave stiffer than it can be.

Increased amphiphilic chitin (reduced starch) in the PLA matrix lowers the tan peak height, which can be related to the restriction of polymer molecule mobility. That is, the chain segments in the neat PLA are unrestricted. On the other hand, T_g_ of biocomposites is somewhat reduced for P8S, P6S2C, and P2S6C, but increased above that of PLA for P4S4C, and P8C, as indicated in Table 3. The variations in the T_g_ values are probably due to disruption in the matrix due to the addition of the starch filler, which is not compatible with the PLA. However, optimum T_g_ was obtained for P4S4C, with an equal percentage of amphiphilic chitin and starch due to the compatibility effect of amphiphilic chitin enhancing miscibility between PLA and starch. Previous studies show reduced T_g_ when degradable fillers are incorporated in PLA [21,43]. This shows that as the filler content increases, the interaction between the filler and the PLA matrix may decrease, and therefore the T_g_ values reduce.

The single relaxation peaks of the DMA analysis can further be double-checked using the Cole–Cole analysis of viscoelastic response [44,45]. Cole–Cole is a method of analysing dielectric relaxation data that involves graphing E″ versus E′, with each point representing one frequency [45,46]. The Cole–Cole approach can be used to investigate the structural changes that occur in biocomposites once fillers are incorporated into the polymer matrix [46,47]. The Cole–Cole complex plane is used to represent dynamic mechanical qualities as a function of temperature and frequency,

(2) $$E^{\prime \prime }=f\left(E^{\prime }\right)$$

**Figure 6:**
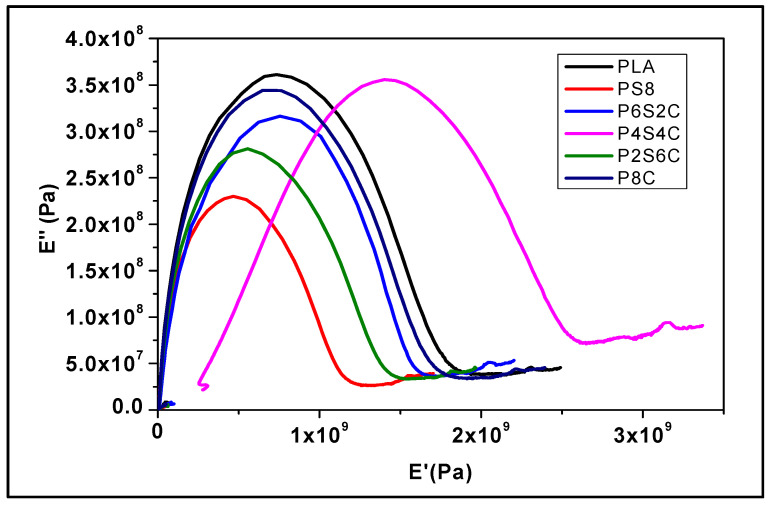
Cole–Cole plots of biocomposites with different starch—amphiphilic chitin content.

Figure 6 illustrates the Cole–Cole curve, which represents the log E″ as a function of the log E′. The shape of the Cole–Cole curve illustrates the nature of the polymeric blend system. A uniform semicircle represents homogeneous polymeric systems, while a deviation from a pure semi-circular form to a skewed arc shape and their centres being well below the horizontal axis are unambiguous indications of dispersed relaxation in the material [48]. Cole–Cole curves are depicted as incomplete semicircles. The shape of the curves indicates that the adhesion between the filler and matrix is relatively excellent. Pothan et al. also observed a similar event [39]. This shows that good miscibility is achieved between the polymeric blend.

The biocomposite shows a well-separated Cole–Cole plot, which means the change in composition variation significantly affects their relaxation time and exhibits distinct dispersions [48]. The deviation in dispersion in this study is most prominent in P4S4C. The characteristic time during which a system relaxes in response to changes in external variables is known as relaxation time. The characteristic time required for a polymer coil to relax from a distorted condition to its equilibrium configuration is referred to here. It is an essential criterion for determining the viscoelastic fluid’s properties. As observed in the Cole–Cole curve, a single relaxation signifies lower disorder when the material is in its viscous state [49]. This means the polymeric blend has formed a uniform mix which is showing good miscibility.

### 3.3. Thermal Properties of Neat PLA and Biocomposites

**Figure 7:**
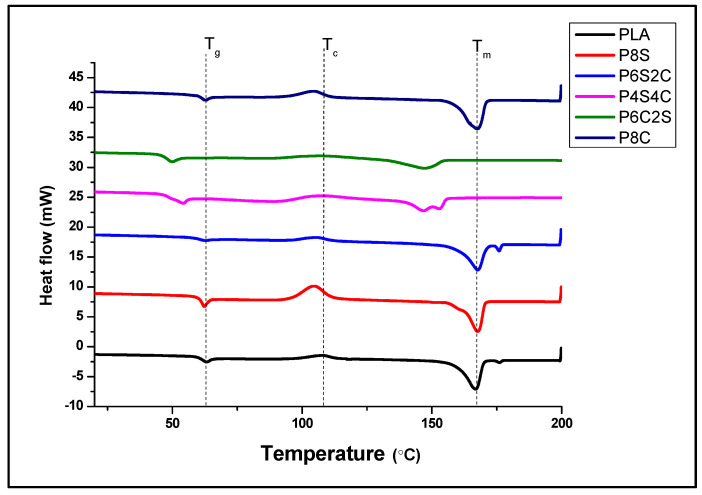
DSC analysis of the neat PLA and biocomposites.

The influence of filler on the melting behaviour of PLA was determined using DSC. Figure 7 illustrates the DSC thermograms, and Table 4 summarises the crystalline and melting temperatures of the neat PLA and biocomposites. The glass transition temperature (T_g_) of PLA is 62.5 °C. The addition of filler to the PLA matrix reduces the T_g_ value slightly, hence increasing the material’s thermal degradability.

The use of natural filler has been reported to enhance the thermal degradability of PLA [27,28]. These findings are in line with TGA conclusions in this study. PLA’s crystallisation temperature (T_c_) drops from 107.5 °C to 106.2 °C as filler is added. This suggests that including a filler with lower crystallinity reduces the matrix’s nucleating ability, which results in a more amorphous biocomposite. The semicrystalline nature of chitin and starch resulted in a sharper crystalline temperature peak, as observed in samples P8S and P8C [50]. However, when the two fillers are used together, the biocomposite crystalline peak seems to disappear, probably due to the lower percentage of the two fillers. 

It was also observed that PLA’s melting point (T_m_) is at 162.3 °C and reduced by adding the two fillers. Biocomposite with both fillers was observed to have two melting peaks. However, this melting peak disappears with either amphiphilic chitin or starch alone as fillers. This could be because when a single filler is employed instead of a double filler, less-perfect crystals have more time to melt and reorganise into crystals with better structural perfection before melting at a higher temperature [51,52]. Furthermore, compared to pure PLA, the low-temperature melting point of biocomposites is lower for P4C4S and P6C2S. Due to the fact that these crystals reorganise and melt at a greater temperature, the melting point of biocomposites shifts somewhat lower. This suggests that adding filler to the PLA matrix can significantly reduce the T_m_ value of PLA.

Mechanical changes are more dramatic than changes in the heat capacity; hence temperature transitions are more visible by DMA than by DSC. As the DMA can detect short-range motion before the glass transition range is reached, it can detect the start of the main chain motion. The T_g_ values obtained from the DSC analysis are comparable to that obtained in the tan delta value in Figure 2. The T_g_ values for PLA, P8S, P6S2C, P4S4C, P2S6C and P8C from the tan delta graph are 73.05 °C, 72.125 °C, 71.84 °C, 71.43 °C, 71.38 °C, and 72.3 °C. The transition temperature values obtained from the tan delta graph show a higher value than that from the DSC result due to the higher sensitivity of the DMA test. However, the trend in the values was similar. A previous study on comparative analysis of T_g_ values from DMA and DSc analysis confirms the difference between the values [53].

**Figure 8:**
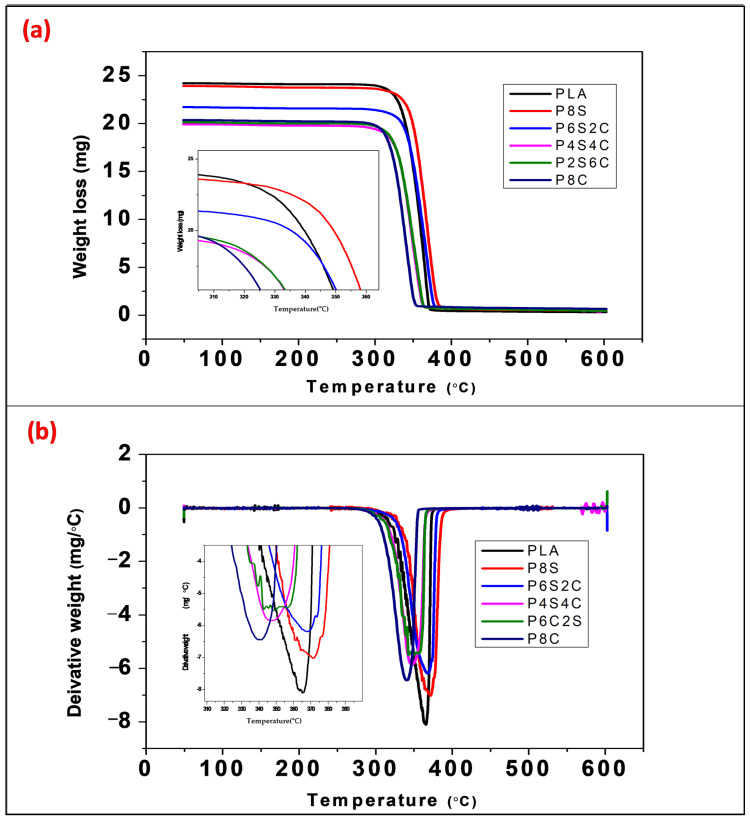
Thermogravimetry plot of the neat polylactic acid and biocomposites (**a**) Thermogravimetric analysis and (**b**) Derivative thermogravimetric analysis.

Thermogravimetric analysis (TGA) was used in this study to assess the thermal stability of polymeric materials. Figure 8a illustrates the TGA curves for the pure PLA and biocomposites. The TGA curves illustrate the single thermal degrading behaviour of either neat PLA or biocomposites when heated from 50 to 600 °C. There is a considerable difference in thermal stability between neat PLA and biocomposites during the first stage, up to 284 °C. All biocomposites fall below the neat PLA. However, the difference in thermal stability is immediately discernible within the key deterioration zone between 284 and 387 °C, where all biocomposites exhibit lower thermal stability than pure PLA. This shows that the addition of starch and amphiphilic chitin probably enhanced the thermal degradation of PLA if used singly or combined. This result is in accordance with Maubane et al. [54]. In their work, the addition of starch enhances the composite’s thermal degradation, which is expected of a biodegradable filler. A similar study on PLA/chitin shows the same trend as observed in this study. Furthermore, the single degradation TGA curve with increasing and decreasing percentages of the fillers probably shows the compatibility effect of amphiphilic chitin.

Figure 8b illustrates the temperature at which the DTG curves reach their maximum for neat PLA and biocomposites, and Table 5 summarises the values. Pure PLA has a greater peak temperature than biocomposites. The DTG temperature falls as the amount of amphiphilic chitin increases and the amount of starch decreases. This demonstrates that the presence of amphiphilic chitin and starch accelerates the thermal breakdown of PLA. Additionally, the combination of amphiphilic chitin and starch accelerated PLA’s heat breakdown. P8S produced the highest peak, while P8C produced the lowest. This indicates that the inclusion of amphiphilic chitin has the potential to accelerate the thermal degradation of PLA more than starch does. Starch’s improved stability may result from the water molecules staying on its surface (hygroscopic), which enhances the scission of PLA’s ester linkages [15,16]. For all samples, the char of the biocomposites is less than that of the clean PLA. This is because filler has lesser thermal stability than the PLA matrix. Natural fillers have been shown to lower the DTG peak temperature of polylactic acid in previous research. This is due to the greater biodegradability of natural fillers than PLA [21,55]. The exception to this is when the filler’s crystallinity outweighs its thermal degradability, as is the case with cellulose nanocrystals [56]. However, this depends on other parameters like the matrix’s composition, the crystal size of the filler, and so forth.

### 3.4. Tensile Properties of Neat PLA and Biocomposite

**Figure 9:**
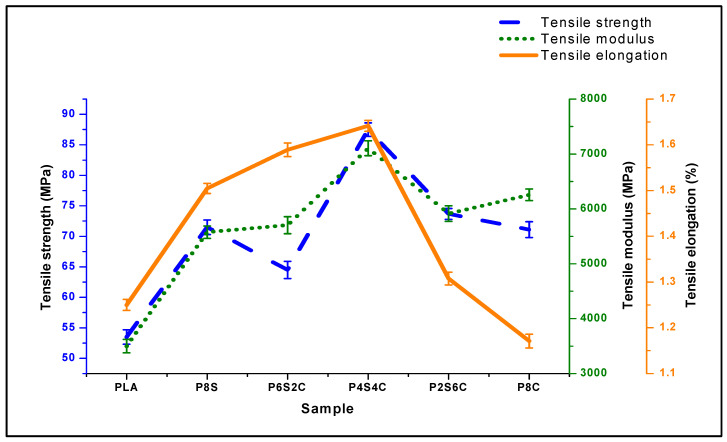
Tensile strength, modulus, and elongation of neat PLA and biocomposite.

The result of the ultimate tensile strength (UTS) test reveals the load-carrying ability of the material. The tensile strength of the polymer biocomposites is shown in Figure 9 (blue coloured).

The biocomposite’s tensile strength was significantly greater than that of the neat PLA (53.5 MPa). However, the graph indicates that PC8 and PC6S2 have lower values due to decreased tensile strength values. The sample PC4S4, with a tensile strength of 87.5 MPa, had the highest tensile strength of the set of samples, followed by PC8 and PC2S6 with only amphiphilic chitin and starch filler. The results indicate that amphiphilic chitin and starch improve the tensile strength of the biocomposite. The higher tensile values of PC4S4 and PC8 justify the modification of amphiphilic chitin. The increase in tensile strength is most likely caused by physical or chemical interaction between the three polymeric components [57]. Tensile strength is almost certainly a good indicator of miscibility [58]. Previously published research has shown that adding starch to the PLA matrix increased its strength and PLA filled with amphiphilic chitin [30,59,60,61]. However, this is not as much as using the combined filler.

The elongation properties of the biocomposite are presented in Figure 9. The elongation value of the biocomposite showed significant improvement compared to the neat PLA. Additionally, the elongation values increased with the amphiphilic chitin content and decreased with starch. The highest elongation value was obtained at P8C. The elongation is observed to increase. This showed that adding amphiphilic chitin enhanced starch’s elongation properties of PLA. This is probably due to the film-forming property of chitin [62]. This showed that the addition of filler also enhanced the elongation of the biocomposite. The literature reported the elongation trend with the PLA blend with chitin and Starch [63,64].

The tensile modulus value in Figure 9 of neat PLA is 3500 MPa. Generally, the modulus of the biocomposite is significantly higher than those of the neat PLA. Similar to the tensile strength results, the modulus of PC4S4 shows the highest strength while that of PC8 is next to it. The lowest among the biocomposite samples was obtained with PS8, with the highest starch percentage. The tensile modulus of biocomposites increased by at least 30% compared with that of the neat PLA. This is quite a significant increase which showed the compatibility between PLA, amphiphilic chitin, and starch.

Table 6 shows the tensile properties of previous studies on Polylactic acid with starch, plasticised starch, amphiphilic chitin, and nano-chitin compared with this study. The result shows a significant increase in the strength and modulus when the ternary blend was used compared with these studies. Additionally, studies on PLA/starch reported a reduction in tensile strength and modulus when plasticiser was used, even though the miscibility between the two polymers was enhanced [16].

Furthermore, the modulus value with an increased percentage of amphiphilic chitin has higher values than those of increased starch. Based on the result, the percentage increase is significant with amphiphilic chitin addition because of the amphiphilic properties of the modified chitin. Additionally, compared with the neat PLA, the combined effect of equal percentage amphiphilic chitin and starch on the tensile modulus of neat PLA is well above those of individual starch or amphiphilic chitin reinforcement. Based on this result, incorporating reinforcement in the PLA matrix enhanced the material’s toughness and reduced its brittleness. Furthermore, the addition of amphiphilic chitin increased the interfacial interaction of the polymeric material, which increased the modulus [67].

### 3.5. Morphological Properties of Neat PLA and Biocomposite

**Figure 10:**
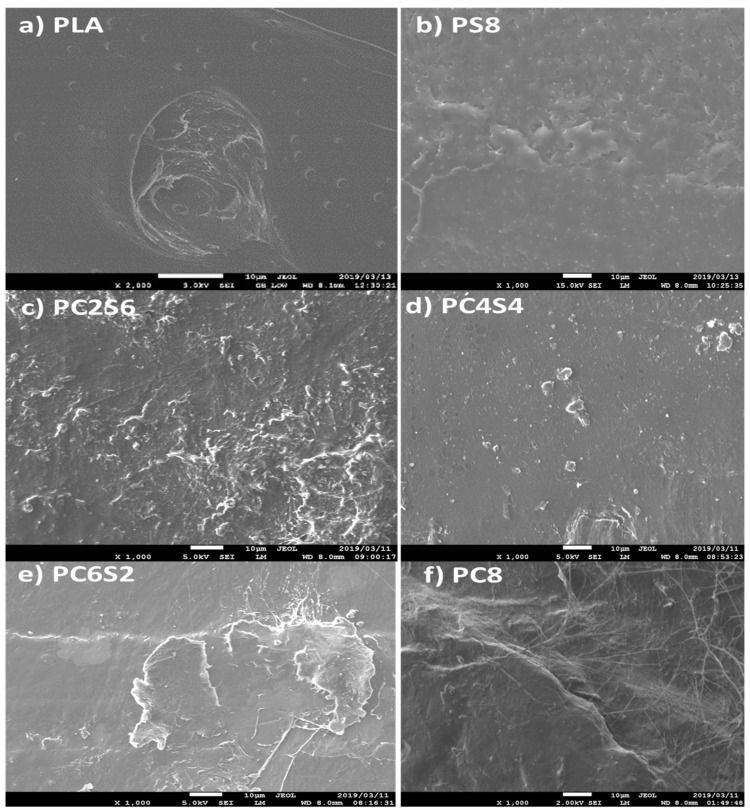
Tensile fractured surface scanning electron microscopy of the biocomposite.

The SEM images demonstrate a smooth surface, indicating the three-polymer blend’s compatibility or adherence. The neat PLA has a plane surface while other biocomposites showed patched filler. The PLA-starch (PS8) sample reveals distinct starch patches in the biocomposite, while PLA-amphiphilic chitin (PC8) almost did not reveal a different coloration from the PLA matrix. This is most likely owing to the chitin modification utilised. The tensile fracture surface morphology of the PLA–amphiphilic chitin–starch composite (Figure 10a–e) exhibits a network of uniformly distributed amphiphilic chitin and starch, with no voids on the shattered surface.

The SEM images reveal an even distribution in the P4C4S sample (Figure 10d), which is likely due to the biocomposite’s equal proportions of amphiphilic chitin and starch. This may account for the sample’s maximum mechanical strength. The dispersion network of the biocomposite samples peaks with a smooth co-continuous morphology with flakes and less defined edges in sample PC62S. SEM images with a cross-hatched network on the surface confirmed improved intermolecular bonding as well [54,68]. Additionally, the biocomposite mix exhibits ridges distributed across its surface rather than a valley crater structure in the shattered surface of the PLA. This is most likely due to the filler’s increased compactness [59]. The morphology demonstrates a high degree of mixing within the blend, which prevents the agglomeration of starch and amphiphilic chitin as considerable edges and wedges with high segmental dispersion were identified, supporting adhesion and interaction.

**Figure 11:**
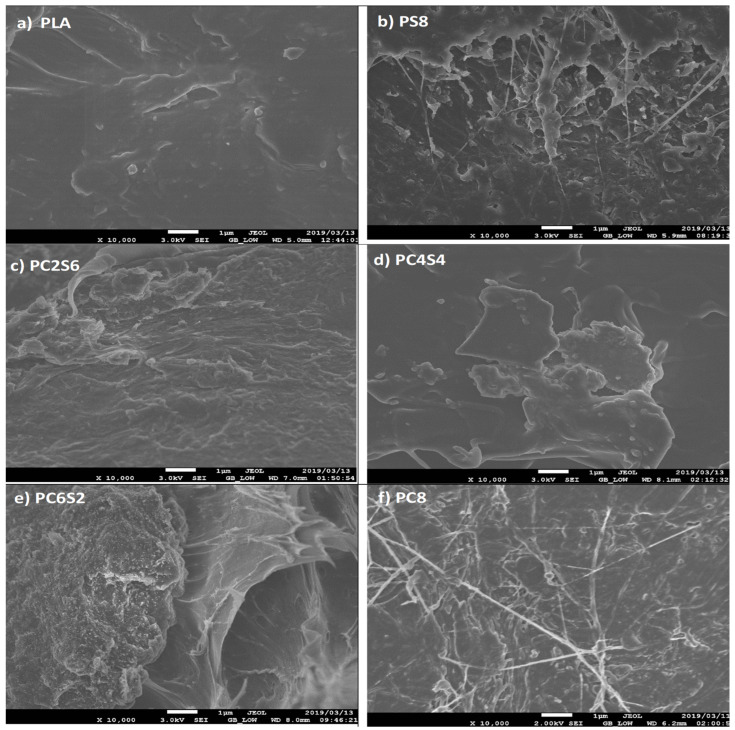
High magnification micrograph of the tensile fractured surface of the biocomposite.

The networks’ bridging effect prevents voids and cracking and enables efficient stress transfer [69]. PC8 (Figure 10f) demonstrated the compatibility effect of amphiphilic chitin by having a smoother surface devoid of white flakes when compared to PLA–amphiphilic chitin–starch (PC6S2, PC4S4, PC6S2) and PLA–starch (PS8) [63]. That is, the inclusion of amphiphilic chitin improves the miscibility of PLA and starch. Chitin (amphiphilic) here has an intrinsic ability to bond with PLA and Starch; therefore, amphiphilic chitin serves as a link that enhances the three PLA–starch compatibilities. This effect is more pronounced in the PLA–amphiphilic chitin–starch biocomposite, which contains an equal amount of amphiphilic chitin and starch with no discernible void. Chitin has been shown to increase the degrading characteristics of PLA, implying a more favourable interfacial interaction between PLA and amphiphilic chitin–starch blends [63]. The interfacial interaction can be confirmed from the high magnification micrograph of the tensile fractured surface of the biocomposite shown in Figure 11.

**Figure 12:**
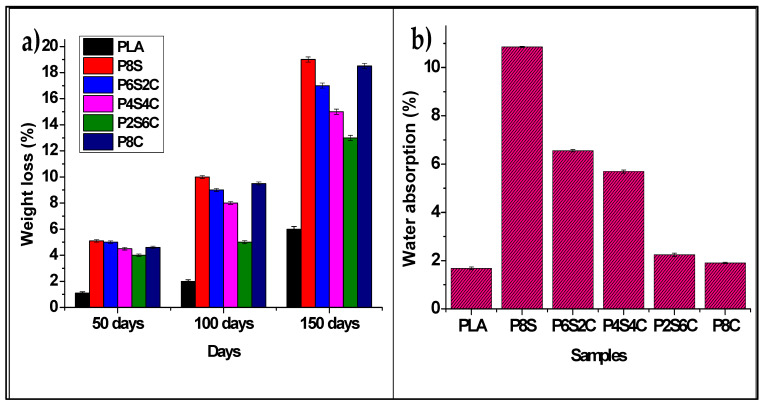
(**a**) Biodegradability and (**b**) water absorption tests of the biocomposite.

The percentage weight loss for the neat PLA and the biocomposites are shown in Figure 12a.

The neat PLA showed 1.1 ± 0.1%, 2.0 ± 0.11%, and 6.0 ± 0.2% weight loss at 50, 100 and 150 days, respectively. The reduction in PLA degradation rate is not spontaneous, probably due to its hydrophobic nature. PLA/starch (P8S) is seen to show the highest degradation percentage of 5.1 ± 0.13%, 10.0 ± 0.13%, and 19.1 ± 0.09%, at 50, 100 and 150 days, respectively. This high degradation could be due to the hydrophilic nature of starch, which absorbed the moisture content in the soil. Furthermore, PLA/amphiphilic chitin (P8S) was also seen to show a higher degradation percentage of 4.6 ± 0.12%, 9.5 ± 0.11%, and 18.5 ± 0.09%, at 50, 100 and 150 days, respectively, compared to the biocomposite with both fillers. The P6S2C, P4S4C, and P2S6C biocomposites showed an enhanced degradation rate than the neat PLA but lower than those with a single filler. Overall, neat PLA has the lowest percentage of weight loss while P8S has the highest.

The weight loss shows that the biodegradability of the biocomposite was significantly improved compared with the PLA. Furthermore, the biocomposite with single filler (P8S and P8C) has a higher weight loss compared with those with double fillers. This is probably due to improved interaction in the microstructure of the biocomposite with two fillers due to the compatibility effect of amphiphilic chitin. The interaction possibly resulted in a stronger bonding which reduced the biodegradable rate. The reduction in biodegradability when a compatibiliser or plasticiser is used has been reported by Phua et al. [70]. The authors reported that better interfacial interaction could reduce weight loss in the biodegradation of biocomposites. PLA degradation can occur in various methods, including molecular hydrolysis, microbiological degradation, thermal photodegradation, and enzymatic degradation [14]. However, enzymatic and microbial breakdown of PLA in the soil is commonly used. PLA has a significant potential to decompose in soil due to many bacterial species. On the other hand, PLA takes a long time to degrade due to its hydrophobic character, as seen in this investigation. The water repellent properties produce a protective layer that inhibits bacteria growth [71].

The water absorption percentage of the neat PLA and the biocomposite is shown in Figure 12b. The neat PLA showed the lowest water absorption while the P8S had the highest. The water absorption shows increased water absorbed with starch and reduction with increased amphiphilic chitin. The increase in water absorption with starch content is probably due to the hydrophilic properties. The reduction with amphiphilic chitin is due to enhanced interfacial interaction and hydrophobic properties due to enhanced interfacial interaction hydrophobic properties acetylated alkyl group substitution [72]. The increase in water absorption with the addition of starch to PLA has been reported by Maubane et al. [54], while the reduction with the addition of chitin has been documented by Nasrin et al. [63].

## 4. Conclusions

Amphiphilic chitin was prepared using acetylation, and the biocomposite was successfully prepared with combined melt extrusion, pelletisation, and compression moulding technique. The storage modulus (E′) is between that of pure PLA and that of biocomposites. The E′ value increases as the amphiphilic chitin content grows and decreases as the starch content increases due to the matrix’s compatibility with the fillers, limiting the PLA matrix’s mobility and deformability. E″ is the modulus of loss that increases as the amphiphilic chitin content increases and decreases as the starch content increases. The damping (tan δ) value reduces as the amphiphilic chitin content increases and increases as the starch content increases. The effect of frequency on the DMA vales of biocomposites demonstrates a significant change in its properties. It is established that the E″ and the T_g_ of all samples increase with increasing frequency due to the delayed relaxing of PLA chains when the high frequency is applied. If the frequency is set high enough, biocomposites would be stiffer. The DSC research reveals that PLA’s T_c_ peak declines with filler incorporation. This shows that the inclusion of both fillers with low crystallinity results in more amorphous biocomposite. All of the biocomposites had a somewhat lower T_g_ than the pure PLA. The TGA data indicate that the T_o_ of biocomposites decreases as the filler content increases. This implies that biocomposites have lower thermal stability than neat PLA, which is attributable to the filler’s lower thermal degradation. According to the findings of this study, a biocomposite composed of 4 wt% percent equal parts amphiphilic chitin and starch is deemed to be the optimal condition. The biocomposite developed in this study has potential application in packaging and biomedical implants due to its green compatibilizer and enhanced mechanical strength.

## Figures and Tables

**Table 1:** Composition variation of the biocomposite.

S/N	PLA (wt%)	Amphiphilic Chitin	Starch (wt%)
Neat PLA	100	0	0
PS8	92	0	8
PC2S6	92	2	6
PC4S4	92	4	4
PC6S2	92	6	2
PC8	92	8	0

**Table 2:** Coefficient of filler effectiveness.

Sample	Coefficient of Composite (*C_comp_*)	Coefficient of Resin (*C_resin_*)	Coefficient of Effectiveness (*C*)
P8S	8.67	14.00	0.62
P6S2C	12.00	14.00	0.86
P4S4C	3.93	14.00	0.28
P2S6C	10.87	14.00	0.78
P8C	12.87	14.00	0.92

**Table 3:** Glass transition temperature values with frequency change.

Sample	Glass Transition Temperature (°C)			
	0.1	1 HZ	5 HZ	10 HZ
PLA	72.8	73.1	73.1	77.6
P8S	72.1	72.1	74.4	76.8
P6S2C	71.8	71.8	74.5	76.4
P4S4C	83.0	83.9	86.3	88.1
P2S6C	72.4	72.3	74.6	77.1
P8C	76.7	76.7	74.4	72.3

**Table 4:** DSC analysis data value.

Sample	T_g_ (C)	T_c_ (C)	T_m_ (C)	
PLA	62.5	107.5	162.3	-
P8S	60.6	106.7	164.3	-
P6S2C	59.8	106.5	163.0	177.3
P4S4C	55.6	-	152.3	155.3
P2S6C	53.3	-	150.2	-
P8C	61.6	106.2	163.1	-

**Table 5:** T.G. onset and DTG peak temperatures of the neat PLA and biocomposites.

Samples	T_o_ (°C)	T_p_ (°C)
PLA	299.8	375.2
P8S	302.1	371.3
P6S2C	286.1	368.8
P4S4C	287.4	349.1
P2S6C	283.6	346.2
P8C	281.3	339.3

**Table 6:** Comparative tensile properties with previous studies.

Composite	Plasticiser	Tensile Strength MPa	Tensile Modulus MPa	Method	Guage Length (mm)	Reference
PLA/starch	none	45.5	1078	Hot press	25	[13]
PLA/starch	Epoxidized Palm Oil	62.5	2750	Hot press	50	[65]
Plasticised PLA/starch	Gelatin	44.7	1175	Hot press	25	[13]
PLA/amphiphilic chitin	none	63	2800	Melt extrusion	50	[66]
PLA/nano chitin	poly(ethylene glycol)	58	3500	Melt extrusion	50	[67]
PLA/amphiphilic chitin/starch	Chloroform	87	7600	Melt extrusion and Compression moulding	50	This study

## Data Availability

Not applicable.
